# Economic and racial disparities of the weather impact on air quality in Brazil

**DOI:** 10.1038/s41598-023-33478-4

**Published:** 2023-04-19

**Authors:** Weeberb J. Requia, Francisco Jablinski Castelhano

**Affiliations:** 1 School of Public Policy and Government, Fundação Getúlio Vargas, Brasília, Distrito Federal, Brazil; 2 Geography Department, Federal University of Rio Grande Do Norte, Natal, Rio Grande Do Norte, Brazil

**Keywords:** Climate sciences, Environmental sciences, Environmental social sciences, Risk factors

## Abstract

Several studies have quantified the air pollution exposure disparities across racial and income groups. However, there is still a lack of investigations assessing disparities related to the impacts of weather on air pollution, which could indicate target air pollution reduction strategies under different climate scenarios. Our study aims to address this gap by estimating the economic and racial disparities of the weather impact on air quality in Brazil between 2003 and 2018. First, we used a generalized additive approach to estimate the weather-related changes in PM_2.5_. This framework derived “weather penalty”, which a positive penalty suggests that an increase in PM_2.5_ was associated with long-term weather changes in the study period. Then, we estimated the population-weighted weather penalty for racial and income groups. Average penalty for the White population (the most-exposed group) was 31% higher than that of the Pardo population (the least-exposed group, mainly people of light brown skin color) in Brazil. In the stratification analysis by region, the Midwest and South were the regions where the black population was the most-exposed group. For the income group, our results indicate that the high-income population group was the most-exposed group in all analyses, including the national and the regional analyses. These findings are somewhat surprising, as previous studies have shown that minority and low-income populations tend to be more exposed to air pollution, than white and higher-income populations. However, our study suggests that disparities in exposure to air pollution may be more complex and nuanced than previously thought. Further research is needed to better understand the underlying drivers of these environmental disparities, and to develop targeted interventions to reduce exposures.

## Introduction

Air pollution is a major environmental risk to health. The World Health Organization (WHO) estimates that every year approximately 4.2 million people die prematurely due to exposure to ambient air pollution^1^. Fine particulate matter of diameters smaller than 2.5 μm (PM_2.5_) itself contributes to approximately two million premature deaths per year, ranking it as the 5th leading cause of worldwide mortality^2^. Even low levels of air pollutants have been associated with substantial health effects^3^.

Air quality is influenced by several factors, including emissions and weather conditions. Air quality standards in the future climate may consider the interaction among these two main factors, given that differences in weather characteristics are altering the advantages of public policies (e.g., emission controls), resulting in additional emissions reductions^4^. In the United States (US), it is estimated that daily variation in meteorology factors can explain up to 50% of PM_2.5_ variability^5^. During 1994–2012, weather-related increases in PM_2.5_ were 0.056 and 0.027 µg/m^3^ per year in the warm and cold seasons in the US, respectively, causing an excess of 770 annual deaths^6^.

Inline with this complex relationship between climate and air pollution, several studies have quantified the exposure disparities across racial and income groups. Here, there are investigations looking at disparities in health risks due to climate change^7^ and investigations focusing on disparities in air pollution^8–10^. These studies have reported that exposure to extreme weather conditions and air pollution are not equitably distributed by race/ethnicity or income. Overall, the literature shows that higher exposures occur for racial/ethnic minority populations and lower-income groups^9,11^. For example, in the US, the average PM_2.5_ concentration for the black population was 13.7% higher than that of the white population and 36.3% higher than that of the Native American population^9^. In another study^10^, it is shown that the disparities in exposure to PM_2.5_ vary across the US states, in which non-Hispanic Black populations have at least 5% higher exposures than average in 63% of states, while Hispanic and non-Hispanic Asian populations have higher exposures in 33% and 26% of states, respectively. In contrast, non-Hispanic White populations did not experience higher exposures in any states. Additionally, 10% increase in the proportion of people with lower socioeconomic status was associated with increases in particulate matter components in the US, including a 20% increase in vanadium and an 18.3% increase in elemental carbon^12^. The economically disadvantaged population in England had NO_2_ levels that were 7.9 µg/m^3^ higher and PM_10_ levels that were 2.6 µg/m^3^ higher compared to the least deprived population^13^. In The Netherlands, the difference in PM_10_ levels was 0.3 µg/m3, while the difference in NO_2_ levels was 6.1 µg/m^3^^13^. In Britain, there was a change in the difference between the mean concentrations for PM_10_ in the most deprived areas and the least deprived areas over time^14^. Specifically, in 2001, the concentration in the most deprived areas was 10.5% higher than in the least deprived areas, but by 2011, this difference had increased to 14.2%^14^. This indicates that the situation in terms of equity had worsened over time.

These exposure disparities studies have contributed to pollution-related regulations, especially in the US, where many of these studies have been conducted. However, while much attention has been paid to the disparities in exposure to air pollution, there has been a lack of research on how weather impacts air pollution, and how these impacts may be distributed unevenly across different racial and economic groups. As climate change continues to alter weather patterns^15–18^, it is becoming increasingly important to understand the intersection between weather, air quality, and social inequalities. By investigating the impact of weather on air quality in different regions and among different population groups, we can identify potential disparities and develop targeted strategies to reduce pollution in a changing climate.

Our study aims to address this gap by estimating the economic and racial disparities of the weather impact on air quality in Brazil. The main novelty of our work is related to the study area—Brazil. The lack of environmental studies in low- and middle-income countries is a critical limitation for the complete understanding of the environmental equity as a social movement to address the unfair exposure of poor marginalized communities worldwide. Brazil faces numerous environmental and social challenges that are strongly correlated with air quality and weather change. First, Brazil is a continental country where there are different types of biomes (e.g., Amazon Forest, Cerrado, Atlantic Forest etc.) with specific natural/anthropogenic air pollution sources and weather changes over space and time. Second, Brazil has a critical challenge related to land use (e.g., agriculture, deforestation etc.), which is also linked to air pollution, weather change and socioeconomic factors. Finally, Brazil has a considerable difference in the quality of health/environment and healthcare across different populations (influencing health/environment equity in negative ways), which is an important determinant of environmental justice.

## Methods

### Weather impacts on air quality (weather penalties)

We used a framework proposed by (Jhun et al. 2015)^6^ to quantify the past weather-related changes in air pollution concentration. This framework derives “weather penalty” by accounting for the differences of the β values among a model adjusted by weather variables and a model unadjusted. Any difference of these models is attributable to the long-term impact of the weather variables. A positive penalty suggests that an increase in air pollution is associated with long-term weather changes. Jhun et al. (2015) used this approach to quantify past weather-related changes (weather penalty) in tropospheric ozone (O_3_) and PM_2.5_ in the US during 1994–2012. Then, further investigations used this same framework in several other analyses, including a study looking at the weather-related impacts in PM_2.5_ elemental concentration in the US^19^, a study that identified where air quality has been impacted by weather changes in the US^20^, a study that quantified the weather-related changes in air pollution in Spain^17^, and a recent study looking at the weather impacts on air quality in Brazil^21^. This recent work in Brazil estimated weather penalties stratified by Brazilian regions (there are five regions in Brazil). In this current study, we estimated weather penalties by municipality, since a fine spatial scale was required to calculate the disparities analyses with better spatial accuracy. There are 5572 municipalities in Brazil, representing the most minor areas considered by the Brazilian political system. We describe below the framework that we used.

We used two datasets, air pollution (PM_2.5_) and weather data. We accessed PM_2.5_ concentration from the Copernicus Atmosphere Monitoring Service (CAMS)-Reanalysis (from the European Centre for Medium-Range Weather Forecasts – ECMWF) for 2003–2018. The data was retrieved at a spatial resolution of 0.125 degrees (approximately 12.5 km), covering Brazil, and a temporal resolution of 6 h, including daily estimates for 00, 06, 12, and 18 UTC—Universal Time Coordinated. We calculated the daily mean concentration for each pollutant. Then, we aggregated air pollution data spatially at the municipality level, considering only the average value of the headquarters of each municipality in Brazil.

Weather data included surface temperature (°C), precipitation (mm), relative humidity (%), and wind speed (m/s). The data were collected from the ERA-Interim model consisting of a global atmospheric reanalysis performed by the ECMWF. The meteorological dataset was also retrieved at a temporal resolution of 6 h and a spatial resolution of 12.5 km. As for PM_2.5_, we calculated the daily means over the entire period of interest for each weather variable, then aggregated the data by the municipality.

As we mentioned above, the weather penalty was derived by the differences of the β values between two models – one model adjusted by weather variables and one model unadjusted. We applied generalized additive models (GAMs) to fit the adjusted and unadjusted models. Both models were controlled for temporal terms, including yearly, monthly, weekday, and daily variation. The adjusted and unadjusted models are described in Eqs. 1 and 2, respectively.

1 $$ Y_{{i,j}}  = \alpha  + \beta _{{adjusted}} year_{{i,j}}  + \gamma month_{{i,j}}  + \delta week\;day_{{i,j}}  + \varepsilon day_{{i,j}} {\text{ }} + {\text{ }}s_{1} \left( {temp} \right){\text{ }} + {\text{ }}s_{2} \left( {ws} \right){\text{ }} + {\text{ }}s_{3} \left( {rh} \right){\text{ }} + {\text{ }}s_{4} \left( {pr} \right){\text{ }} + {\text{ }}e_{{i,j}}  $$

2 $$ Y_{{i,j}}  = \alpha  + \beta _{{unadjusted}} {\text{ }}year_{{i,j}}  + \gamma month_{{i,j}}  + \delta week\;day_{{i,j}}  + \varepsilon day_{{i,j}} {\text{ }} + {\text{ }}e_{{i,j}}   $$

where* Y* represents the daily concentration of PM_2.5_ in the municipality *i* on date *j*; *α* is the intercept of the GAM model; β_unadjusted_ and β_adjusted_ represent the linear weather-unadjusted and adjusted PM_2.5_ trends, respectively, expressed in μg/m^3^ per year; $\gamma$, δ, and Ɛ are the vectors of coefficients that explain monthly, weekday, and daily variability within the time series, respectively; *e* are normal residual errors with homoscedastic residual variance; and *s1, s2, s3* and *s4* are the default smoothing spline functions from the mgcv R package, that take into account the nonlinear relationships between daily concentration of PM_2.5_ and weather variables, including temperature (temp), wind speed (ws), relative humidity (rh), and precipitation (prec), respectively in the weather-adjusted model (Eq. 1).

Then we used the ${\beta }_{adjusted}$ and ${\beta }_{unadjusted}$ values to quantify past weather-related changes (“weather penalty”, expressed in µg/m^3^ per year) in PM_2.5_. We derived the weather penalties for each municipality by obtaining the differences between ${\beta }_{unadjusted}$ and ${\beta }_{adjusted}$ (${\beta }_{unadjusted}$ − ${\beta }_{adjusted}$). While the weather impact is incorporated into the unadjusted trends (Eq. 2), the control by weather variables in model 1 removes the impact of inter-annual weather variation on PM_2.5_ trends. Therefore, we considered that any differences between the unadjusted and weather-adjusted trends are entirely attributable to long-term weather changes. A positive penalty (${\beta }_{unadjusted}$ > ${\beta }_{adjusted}$) suggests that an increase in PM_2.5_ is associated with long-term weather changes between 2003 and 2018. On the other hand, a negative penalty indicates that variation in weather variables over the study period was associated with decreased pollution.

Finally, we applied bootstrap analysis to compute the confidence intervals for the coefficients estimated above. The bootstrap was based on randomized subsets (pseudo-datasets) of the input dataset that accounted for serial correlation structures among the observations. We created 1000 pseudo-datasets for each municipality. Then, for each pseudo-dataset, we applied the same models described in Eqs. 1 and 2 (adjusted and unadjusted, respectively). Then, we estimated standard error by obtaining standard deviation from the 100 estimates in the bootstrap analysis.

### Disparities analyses

The disparities analyses were divided into three steps, including (i) the calculation of the population-weighted weather penalty, (ii) the calculation of the difference between exposure for the most-exposed group versus the least exposed group, and (iii) the estimation of the weighted Gini coefficients. All these analyses were performed on a national and regional scale.

#### Population-weighted weather penalty

The population-weighted weather penalty was calculated for two groups—racial and income. For both groups, we used population census data provided by the Brazilian Institute of Geography and Statistics—BIGS (https://www.ibge.gov.br/en/). The BIGS classifies the race group into four groups, including white, black, *pardo* (mainly used to refer to the people of light brown skin color), and *amarelo* (direct translation to English, it means “yellow”; technically, according to the BIGS, it refers to Asian people). These race categories were the option available chosen by the participants of the census. The national population-weighted weather penalty for racial group *k* was calculated as:

3 $$ \overline{WP}_{k} = \frac{{\mathop \sum \nolimits_{j = 1}^{n} WP_{j} P_{k,j} }}{{\mathop \sum \nolimits_{j = 1}^{n} P_{k,j} }} $$

where ${\overline{WP} }_{k}$ is the national population-weighted Weather Penalty (*WP*) for racial group *k* (White, Black, Pardo, or Asian), measured in µg/m^3^; ${WP}_{j}$ is the weather penalty for municipality *j*; ${P}_{k,j}$ is the number of people in racial group *k* living in the municipality *j*; and *n* is the number of municipalities in Brazil. For the income groups, the population-weighted weather penalty was calculated as:

4 $$ \overline{WP}_{i} = \frac{{\mathop \sum \nolimits_{{j = 1 \left( {j \in i} \right)}}^{n} WP_{j} P_{j} }}{{\mathop \sum \nolimits_{{j = 1 \left( {j \in i} \right)}}^{n} P_{j} }} $$

where ${\overline{WP} }_{i}$ is the national population-weighted Weather Penalty (*WP*) for income group *i*, also, measured in µg/m^3^. Here, we accounted for two income groups, including the group categorized as low income (< quartile 25th) and the group defined as high income (> quartile 75th); ${WP}_{j}$ is the weather penalty for municipality *j*; ${P}_{j}$ is the total population of municipality *j*. Note that the summation occurs only across municipality *j* belonging to income group *i*. Thus, *n* represents these municipalities.

#### Difference between exposure for the most-exposed group versus the least exposed group

In the second stage, we calculated the exposure disparity based on three metrics, absolute disparity, percent difference, and relative disparity (ratio). The absolute disparity was calculated as the difference between the exposure for the most-exposed group (racial and income) and the exposure for the least-exposed group (racial and income). For example, considering that black population were more exposed to weather penalties than the white population (${\overline{WP} }_{black}>{\overline{WP} }_{white}$), thus, the absolute disparity would be calculated as ${\overline{WP} }_{black}-{\overline{WP} }_{white}$. This metric is linked to exposure-specific health impacts^22^. For the second metric, still considering the example mentioned above (${\overline{WP} }_{black}>{\overline{WP} }_{white}$), the percentage difference would be calculated as *[(*${\overline{WP} }_{black}-{\overline{WP} }_{white}$*)/national mean weather penalty]* × *100%*. Finally, the relative disparity would be calculated as ${\overline{WP} }_{black} / {\overline{WP} }_{white}$. The metrics of percent difference and relative disparity are used to quantify disproportionality in exposure burdens^22^.

#### Estimation of the weighted Gini coefficients

Note that the metrics described in the previous topic are based on population-weighted mean weather penalty exposures. A limitation of these metrics is that the disparities are not calculated across the full weather penalty distribution. Therefore, to address this limitation in a way that we can verify the consistency of our primary metrics (mentioned in the previous section), in this third stage, we calculated the inequality metric considering the full weather penalty exposure distribution by estimating weighted Gini coefficients for each racial group and for the overall population. The weighted Gini coefficient was calculated using the *weighted.gini* function in the R package “acid”. In this function, we used as inputs the weather penalties and the population for the racial group (and total population) for each municipality.

### Ethical approval

All experiments were performed in accordance with relevant guidelines and regulations.

## Results

### PM_2.5_, weather penalties, racial groups, and income in Brazil

**Figure 1:**
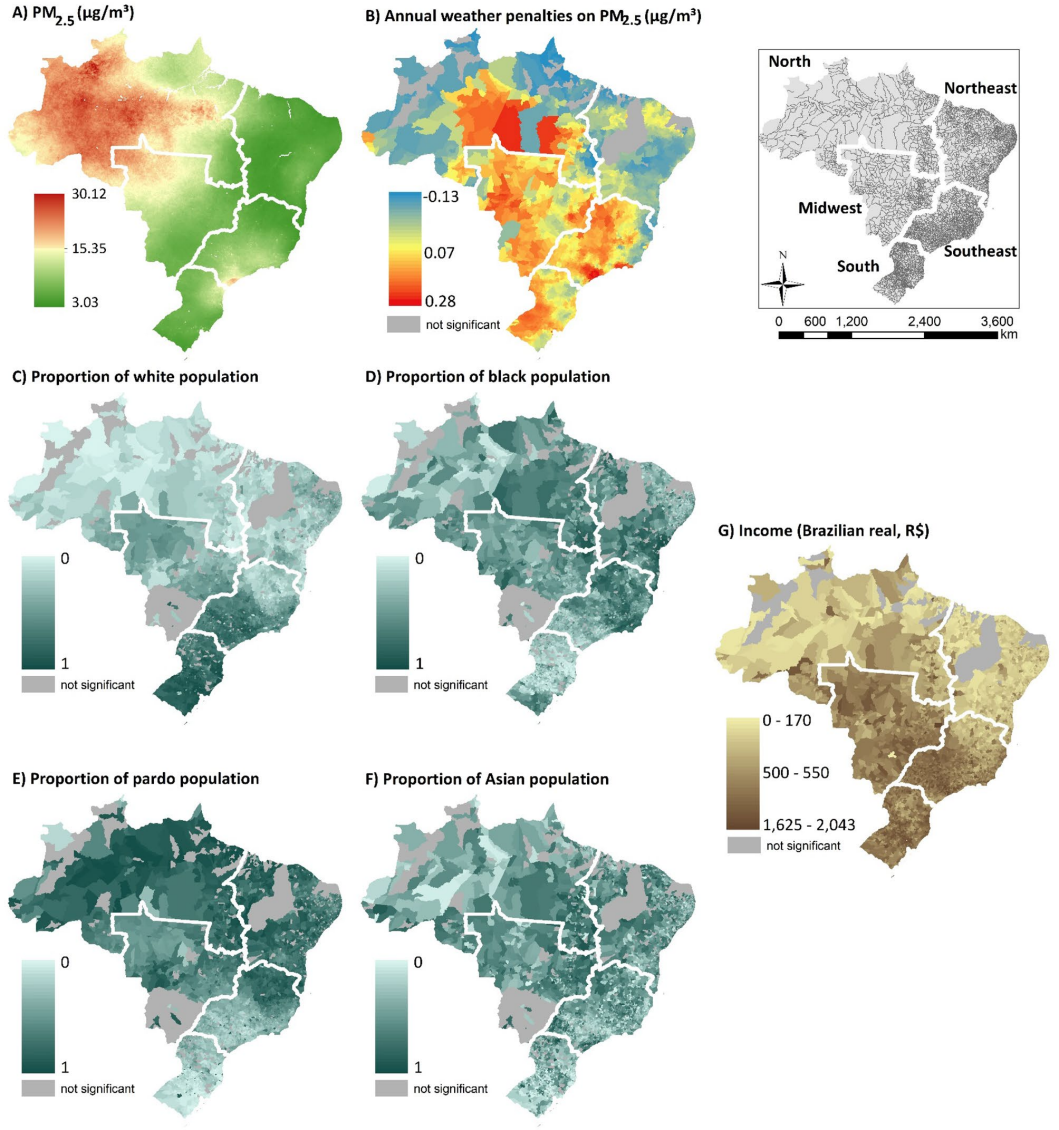
Spatial distribution of PM_2.5_ concentration (spatial resolution of 1 × 1 km), annual weather penalties on PM_2.5_ (by municipalities), population by race groups (population in the racial group/total population, by municipalities), and income (by municipalities). *Note 1*: “not significant” represents the municipalities with non-significant penalties. *Note 2*: the top right map shows Brazil's regions and municipalities. *Note 3*: This figure was created by R version 4.1.3. 'https://www.r-project.org/'.

Figure 1 shows the nationwide concentrations of PM_2.5_, weather penalties, population by race groups, and income in Brazil. North is the region with the highest PM_2.5_ concentration, especially in the Amazon region (Fig. 1, map A), where a large area has a concentration of about 30 µg/m^3^. This spatial distribution of PM_2.5_ in Brazil may be due to the mix of chemical species in the air particulates in the Amazon region, such as nitrate (mostly biogenic productions), ammonium sulfate (biogenic origin from the forest), organics, mineral dust mixed with sea salts (probably during long-range transatlantic transport from the Sahara Desert), and elemental carbon (anthropogenic origin)^23^.

For the weather penalties on PM_2.5_ (Fig. 1, map B), most of the Brazilian municipalities (a total of 5086) had positive penalties, indicating that an increase in PM_2.5_ was associated with long-term weather changes between 2003 and 2018 in those municipalities. We suggest that this positive weather penalty includes the direct (e.g., photochemical reactions, transport by the wind of air pollutants or their precursors, changes in precipitation patterns) and indirect (e.g., deforestation) impacts of meteorological conditions, and those of other weather events occurring in the region (e.g., transport of cold, dry, air mass). The highest annual penalty was 0.28 µg/m^3^, indicating that if weather variables had remained constant in the 16-year period of study in the municipalities with this penalty, PM_2.5_ would have decreased by 4.48 µg/m^3^ (0.28 × 16). On the other hand, 93 municipalities had negative penalties, suggesting that air quality was improved in these municipalities due to weather variation between 2003 and 2018. A total of 70 municipalities presented statistically insignificant penalties, which the 95% confidence interval included the value 0. Note that these municipalities with non-significant penalties were removed in further analyses (disparities analyses). We can observe that the highest penalties were distributed over the municipalities in the Midwest, Southeast, and South. Even with high concentrations of PM_2.5,_ in the North and Northeast, only a few municipalities had positive penalties.

Regarding the distribution of population stratified by race (Fig. 1, maps C, D, E, and F), the high proportion of the white population is intensely distributed over the municipalities in the South region and part of the Southeast region (Fig. 1, map C), while a high proportion of black (Fig. 1, map D), pardo (Fig. 1, map E), and Asian (Fig. 1, map F) population are distributed over the other regions. For income, high-income municipalities are clustered in the Midwest, Southeast, and South region. Northeast and North regions have the lowest-income municipalities (Fig. 1, map G).

**Figure 2:**
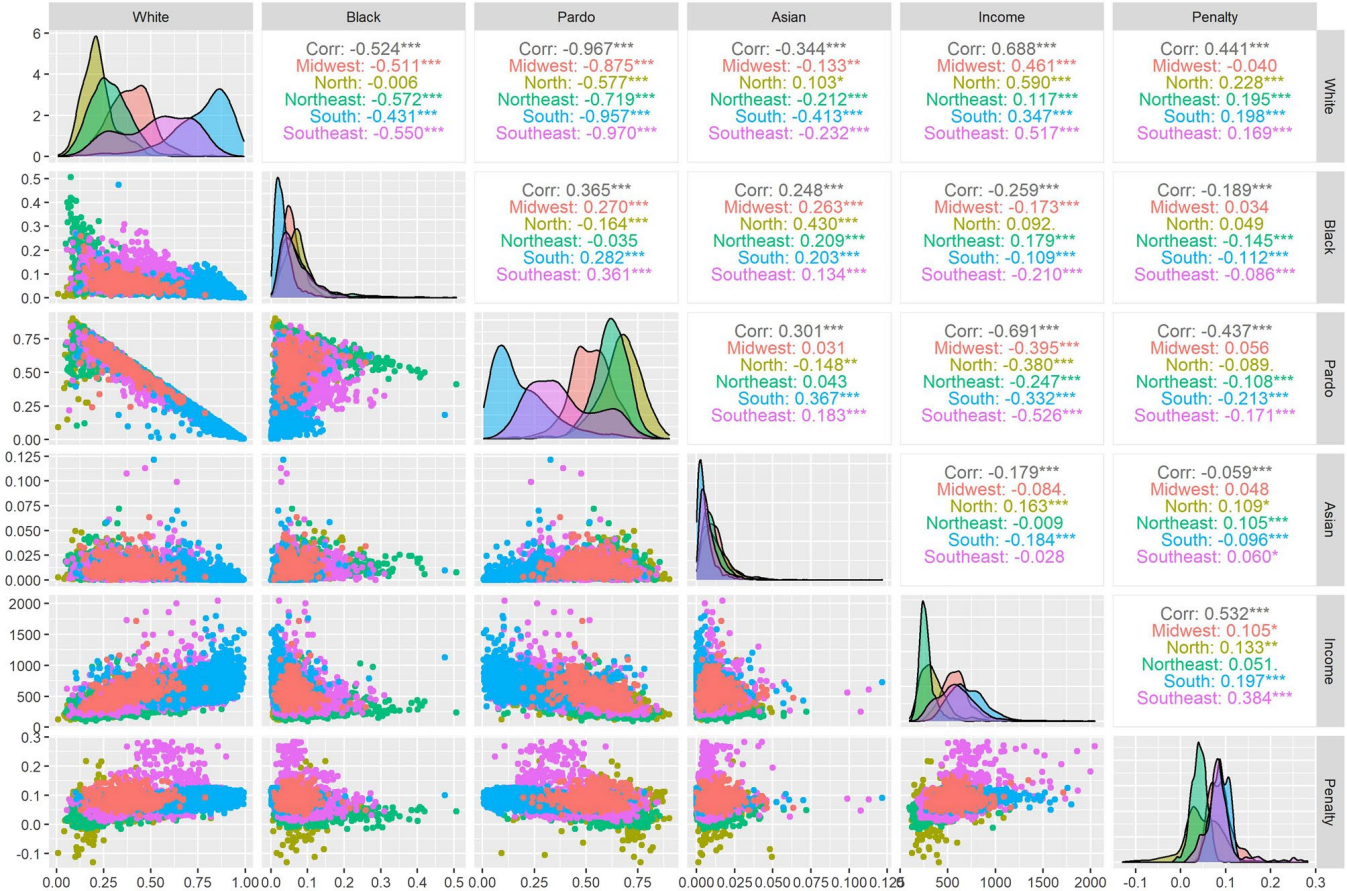
Correlation matrix, density plots, and scatter plots for weather penalty, population by race groups, and income stratified by regions. *Note*: *indicates the significance level for the Spearman coefficients; “Corr” is the correlation at national level.

Figure 2 summarizes the distribution (with density plots, scatter plots, and correlation matrix) of the weather penalty, population by race groups, and income stratified by regions. Figure 2 is a supplement to Fig. 1. For example, we can observe that the penalties have a moderate correlation (r = 0.53) with income in Brazil. In the subgroup analysis, Southeast had the highest correlation coefficient (r = 0.38). The highest correlation between the penalties and the race groups was observed for the white population (r = 0.44) (Fig. 2).

### Racial and economic disparities

After accounting for the population-weighted average penalty for the racial population group, we found that the average penalty for the white population was 31.60% higher than that of the pardo population in Brazil. In the stratification analysis by region, the results varied substantially. Asians were the most-exposed group in the North and Southeast regions, whites were the most-exposed in the Northeast and region, and the blacks were the most-exposed in the Midwest and South regions. Southeast had the highest difference, indicating that the average penalty for the Asian population in this region was 24.85% higher than that of the black population (Table 1).

**Table 1:** Difference between the population-weighted weather penalty for the most-exposed group (racial and income group) versus the least-exposed group (Main analysis).

Region	Group considered in the difference	Most-exposed group (WP)	Least-exposed group (WP)	Absolute disparity	% difference	Relative disparity
National	Racial group	White (WP = 0.11)	Pardo (WP = 0.09)	0.023	31.60	1.26
	Income group	Q75 (WP = 0.12)	Q25 (WP = 0.04)	0.083	115.16	3.05
North	Racial group	Asian (WP = 0.06)	Black (WP = 0.05)	0.007	14.02	1.14
	Income group	Q75 (WP = 0.06)	Q25 (WP = 0.04)	0.020	41.64	1.54
Northeast	Racial group	White (WP = 0.04)	Black (WP = 0.03)	0.010	23.67	1.30
	Income group	Q75 (WP = 0.04)	Q25 (WP = 0.04)	0.004	9.03	1.10
Midwest	Racial group	Black (WP = 0.11)	White (WP = 0.11)	0.003	2.90	1.02
	Income group	Q75 (WP = 0.11)	Q25 (WP = 0.09)	0.023	26.60	1.26
Southeast	Racial group	Asian (WP = 0.17)	Black (WP = 0.14)	0.022	24.85	1.15
	Income group	Q75 (WP = 0.16)	Q25 (WP = 0.07)	0.087	99.10	2.18
South	Racial group	Black (WP = 0.09)	Asian (WP = 0.08)	0.008	9.15	1.10
	Income group	Q75 (WP = 0.09)	Q25 (WP = 0.08)	0.009	10.91	1.12

*Note 1*: population-weighted weather penalty (WP), third quartile of the income distribution (Q75), first quartile of the income distribution (Q25).

For the income group, our results indicate that the high-income population group (income > third quartile of the income distribution) was the most-exposed group in all analyses, including the national and the regional analyses. Overall, the percentage difference for the income group was higher than that of the racial group. For example, we found that the average penalty for the high-income group was 115.16% higher than that of the low-income group in Brazil (Table 1).

In Table 2, we show the supplemental inequality metric considering the Gini coefficients for weather penalties. At the national level, the black group had the higher Gini coefficient (0.41), while the white, Asian, and pardo groups the coefficient were 0.33, 0.39, and 0.40, respectively. Considering the total population, the North region presented the highest Gini coefficient (0.48). The Midwest and South regions had the lowest coefficient, 0.15 (Table 2).

**Table 2:** Gini coefficients for the total population and racial group for weather penalties.

Region	Total population	White group	Black group	Asian group	Pardo group
National	0.37	0.33	0.41	0.39	0.41
Midwest	0.15	0.16	0.15	0.15	0.15
North	0.48	0.46	0.50	0.43	0.48
Northeast	0.27	0.24	0.42	0.28	0.27
South	0.15	0.15	0.15	0.16	0.16
Southeast	0.27	0.27	0.27	0.25	0.28

## Discussion

**Figure 3:**
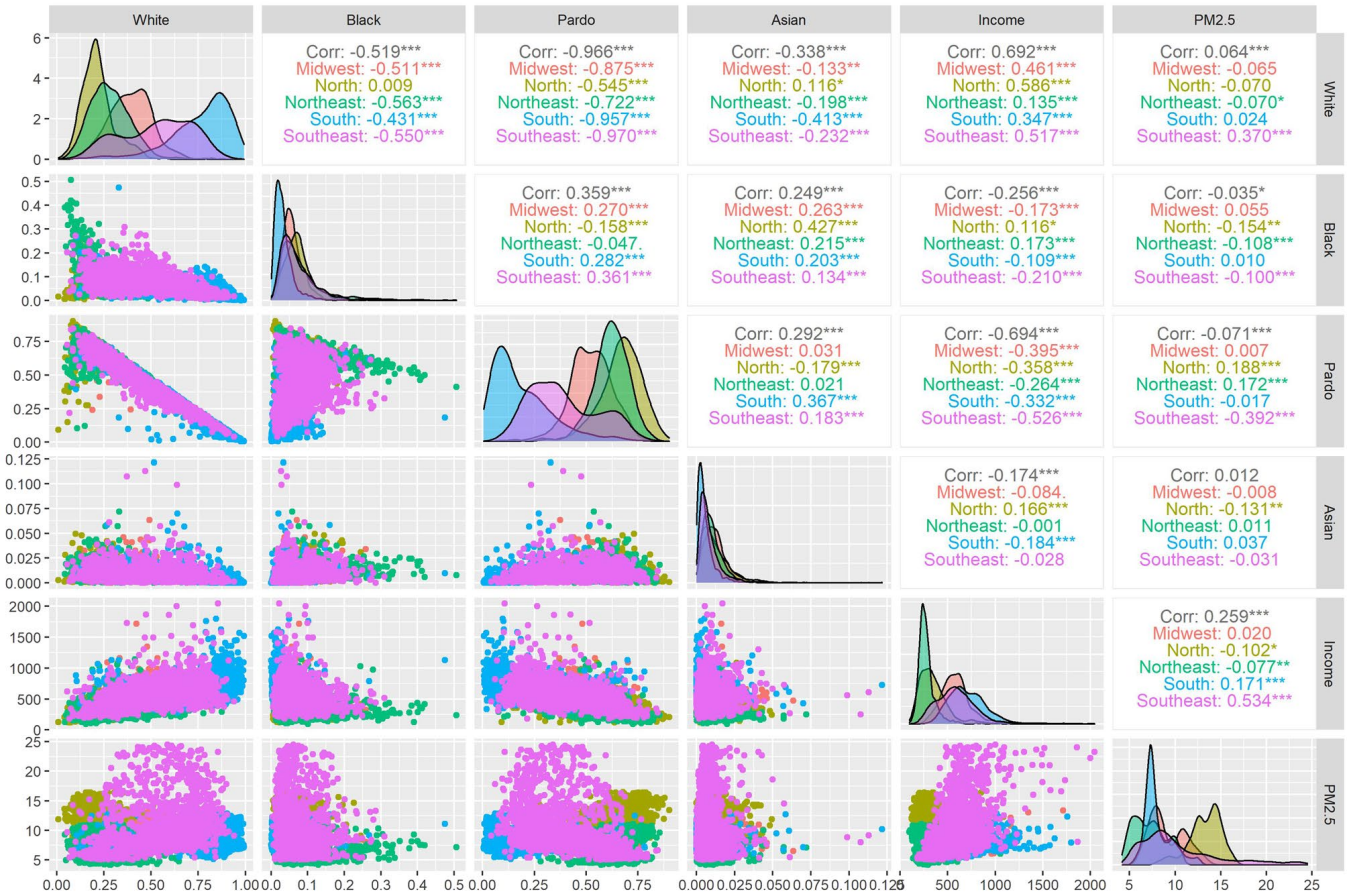
Correlation matrix, density plots, and scatter plots for PM_2.5_, population by race groups, and income stratified by regions. *Note*: *indicates the significance level for the Spearman coefficients; “Corr” is the correlation at national level.

Our findings suggest that the high-income groups were the most exposed income groups. For racial groups, our findings varied by Brazilian region. It is difficult to compare our results with the literature since we are unaware of previous studies looking at disparities in the weather's impact on air quality. The majority of the prior studies assessed disparities in air pollution exposure. A comparison here can provide a sense of the underlying drives of the observed disparities with similar interpretations. However, this assessment should be done with caution, considering the specificity of each exposure variable. To facilitate this comparison purpose, we have applied an additional analysis accounting for disparities in exposure to PM_2.5_ (same as the literature) instead of weather penalties. These additional analyses are shown in Fig. 3 and Table 3. We suggest that the discussion with the literature will be more feasible if we take together both the primary (disparities in weather penalties) and the additional analysis (disparities in PM_2.5_).

**Table 3:** Difference between the population-weighted PM_2.5_ for the most-exposed group (racial and income group) versus the least-exposed group.

Region	Group considered in the difference	Most-exposed group (WP)	Least-exposed group (WP)	Absolute disparity	% difference	Relative disparity
National	Racial group	Asian (WP = 13.43)	Pardo (WP = 12.04)	1.389	15.27	1.12
	Income group	Q75 (WP = 14.58)	Q25 (WP = 8.55)	6.039	66.39	1.71
North	Racial group	Pardo (WP = 14.50)	Asian (WP = 14.27)	0.225	1.69	1.02
	Income group	Q75 (WP = 14.81)	Q25 (WP = 13.90)	0.914	6.88	1.07
Northeast	Racial group	Asian (WP = 7.67)	Black (WP = 7.50)	0.171	2.38	1.02
	Income group	Q75 (WP = 7.8)	Q25 (WP = 7.74)	0.055	0.77	1.01
Midwest	Racial group	Asian (WP = 11.15)	Pardo (WP = 10.97)	0.176	1.98	1.02
	Income group	Q75 (WP = 11.55)	Q25 (WP = 10.01)	1.537	17.34	1.15
Southeast	Racial group	Asian (WP = 17.96)	Pardo (WP = 16.07)	1.893	18.22	1.12
	Income group	Q75 (WP = 18.29)	Q25 (WP = 7.14)	11.149	107.32	2.56
South	Racial group	Black (WP = 9.34)	Pardo (WP = 8.83)	0.513	6.36	1.06
	Income group	Q75 (WP = 9.46)	Q25 (WP = 7.69)	1.766	21.88	1.23

*Note 1*: population-weighted weather penalty (WP), third quartile of the income distribution (Q75), first quartile of the income distribution (Q25).

For the income population group, our findings indicate a substantial similarity between the population-weighted weather penalty (Table 1) and population-weighted PM_2.5_ (Table 3). Both analyses showed that the high-income groups were the most exposed. In contrast, for racial groups, the were some differences.

Regarding the weather's impact on air quality (Table 1), we found that white group was the most exposed group at the national scale, with 31% more exposure compared to the Pardo population, the least exposed group. These findings are somewhat surprising, as previous studies have shown that minority and low-income populations tend to be more exposed to air pollution, than white and higher-income populations^12,24–26^. The disparities in the weather's impact on air quality varied across different regions and ethnic groups in Brazil. For example, in the North region, Asians were the most exposed group, while in the Northeast region, Whites were the most exposed group. In the Midwest and South regions, Blacks were the most exposed group, and in the Southeast region, Asians were the most exposed group.

On the other hand, we found that the Asian group was the most exposed group to PM_2.5_ (population-weighted PM_2.5_) at the national scale, with 15% more exposure compared to the Pardo population, the least exposed group (Table 3). Similar to the weather's impact on air quality, disparities in PM_2.5_ exposure also varied across different regions and ethnic groups in Brazil. For example, in the North region, Pardo were the most exposed group to PM_2.5_, while in the South region, Blacks were the most exposed. In the Northeast, Midwest, and Southeast regions, Asians were the most exposed group to PM_2.5_.

In the USA, although low-income group and black communities have been identified as the group most exposed to PM_2.5_ in several studies^12,24–26^, these results may not be homogeneous over space and time, in some regions and periods white communities and high-income group are the most exposed to PM_2.5_^9,10^. For example, while in the Mid-West and Mid-Atlantic regions in the USA the white and Asian populations are exposed to higher levels of PM_2.5_ than the black population, in the Southeast region, the black populations are exposed to the highest level of PM_2.5_^9^. Among the American states, it is shown that the disparities in exposure to PM_2.5_ vary substantially, with non-Hispanic Black populations experiencing at least 5% higher exposures than the national average in 63% of states, while Hispanic and non-Hispanic Asian populations have higher exposures in 33% and 26% of states, respectively. Non-Hispanic White populations, on the other hand, did not experience higher exposures in any state^10^. In China, characteristics of the exposure and inequality of PM_2.5_ was different from the USA and similar to our study, which higher income subgroup and majority ethnic group have the most significant exposure to PM_2.5_^27,28^. A review study in the World Health Organization European Region accessed 31 articles and found mixed results on the social inequalities in exposure to ambient air pollution^29^. In Australia, overall, it is estimated that socio-economic disadvantage populations may be inequitably exposed to PM_2.5_, but this link is complex with may non-linear relationships^8^.

We suggest that part of our findings may be explained by Brazil's complex environmental and socio-demographic conditions. We must consider that Brazil has different types of biomes (Amazon Forest, Cerrado, Atlantic Forest, Caatinga, Pampa, and Pantanal) that are strongly correlated with weather^30,31^ and land use^32,33^—an essential proxy of air pollution^34,35^. For example, the Caatinga biome (mostly located in the Northeast region) has become warmer and dryer in the last years^33,36^ and there has been a considerable increase in agricultural activities and pasture area^32,33^. Regarding the socio-demographic conditions, according to the last census, the highest percentage of the black population in Brazil is in the Northeast (9.5%) and Southeast (7.9%), while the South has the lowest (4.1%). Paradoxically, Northeast is the regions with the lowest average income and Southeast has highest average income. While the Southeast concentrates the largest urban areas and industries, the Northeast region has the highest rural population in Brazil, with over a quarter of its population living in the countryside.

Considering these conditions in the Northeast, overall, we can assume that the municipalities identified with positive penalties are those with high-income populations due to the intensive agricultural activity and pasture area. On the other hand, compared to the Northeast, the Midwest municipalities (mostly with the Cerrado biome) and South (mainly with the presence of the Pampa biome) had the highest weather penalties on PM_2.5_. Both regions have a substantial number of municipalities with high-income, over 80% of urban population, but while the Midwest has a heterogeneous race distribution, the South region has a substantial proportion of the white population. These regions have a mix of land use classes, including large urban areas (comprising massive industrial areas), pasture, agricultural activities (e.g., soybean, corn, and sugarcane crops), and forest areas and very similar average incomes. Note that these were the only regions in our analyses where the black population was the most-exposed group to weather penalties on PM_2.5_.

Our study has limitations. First, the measurement of weather variables and PM_2.5_ (input datasets to estimate the weather penalties on PM_2.5_) was based on satellite remote sensing. This may result in a misclassification error and non-differential misclassification. However, a recent study compared the weather data reported by ECMWF with weather station data and found comparable performance^37^. Regarding the PM_2.5_ concentrations, it was relied on aerosol optical depth (AOD) data and validated with ground observations of the Aerosol Robotic Network (AERONET). There are over 500 AERONET stations worldwide measuring spectral Aerosol Optical Depth (AOD) with ground-based sun photometers. Among those AERONET stations, about 27 stations are in Brazil. Considering the measurement of these stations, the CAMS global model was validated, indicating good performance in Brazil ^38^. Also, our analysis was based on a municipal scale, which can mask the relationship between weather penalties and income/race data, especially where the significant variation of weather penalties may occur within municipalities. Another limitation of our study is that we did not estimate disparities over time. Some studies have reported substantial variation in disparities over time^14^. For example, in Britain, there was a shift in the difference between the average PM_10_ concentrations in the most deprived and least deprived area. In 2001, the concentration in the most deprived areas was 10.5% higher than in the least deprived areas; in 2011, this difference had grown to 14.2%^14^. In addition,, our study does not indicate causal aspects of weather penalties and economic/racial disparities in Brazil. This is only a descriptive study. Finally, our findings are based on data from a census, which may have certain limitations that could impact our results. One potential limitation is the sample size for each racial category, which varied widely. While we attempted to address this by conducting separate analyses for each group, it is possible that our results may not fully capture the experiences of all individuals in each racial category. Additionally, it is important to consider that the census data only includes self-reported race, which may not fully capture the complexity and nuance of an individual's racial identity or experiences.

## Conclusions

To our knowledge, this is the first study providing an analysis of the economic and racial disparities of the weather impact on air quality. In Brazil, this is the first study assessing national disparities for some environmental exposure variables.

We have shown here that the high-income group and mostly the whites were the most exposed population group. The effect size of this disparities varies significantly among the Brazilian regions. These findings differ dramatically from most of the existing environmental justice literature, including the results reported by recent investigations in the United States. On the other hand, our findings are similar to previous investigations in China.

Further studies in Brazil and other low- and middle-income countries are necessary to establish a body of evidence jointly with our findings. Specifically in Brazil, further investigations could focus on human health conditions and others economic indicators. This literature body can inform the strategies for reducing air pollution (e.g., adopt vehicle emission standards, cutting emissions from power plants, controlling wildfires etc.) under climate scenarios in order to decrease weather penalties and the relative disparities.

## Data Availability

The datasets generated during and/or analysed during the current study are available from the corresponding author on reasonable request.
